# Detrended Partial-Cross-Correlation Analysis: A New Method for Analyzing Correlations in Complex System

**DOI:** 10.1038/srep08143

**Published:** 2015-01-30

**Authors:** Naiming Yuan, Zuntao Fu, Huan Zhang, Lin Piao, Elena Xoplaki, Juerg Luterbacher

**Affiliations:** 1 Chinese Academy of Meteorological Science, Beijing, 100081, China; 2 Department of Geography, Climatology, Climate Dynamics and Climate Change, Justus Liebig University Giessen, Senckenbergstrasse 1, 35390 Giessen, Germany; 3 Lab for Climate and Ocean-Atmosphere Studies, Dept. of Atmospheric and Oceanic Sciences, School of Physics, Peking University, Beijing, 100871, China

## Abstract

In this paper, a new method, detrended partial-cross-correlation analysis (DPCCA), is proposed. Based on detrended cross-correlation analysis (DCCA), this method is improved by including partial-correlation technique, which can be applied to quantify the relations of two non-stationary signals (with influences of other signals removed) on different time scales. We illustrate the advantages of this method by performing two numerical tests. Test I shows the advantages of DPCCA in handling non-stationary signals, while Test II reveals the “intrinsic” relations between two considered time series with potential influences of other unconsidered signals removed. To further show the utility of DPCCA in natural complex systems, we provide new evidence on the winter-time Pacific Decadal Oscillation (PDO) and the winter-time Nino3 Sea Surface Temperature Anomaly (Nino3-SSTA) affecting the Summer Rainfall over the middle-lower reaches of the Yangtze River (SRYR). By applying DPCCA, better significant correlations between SRYR and Nino3-SSTA on time scales of 6 ~ 8 years are found over the period 1951 ~ 2012, while significant correlations between SRYR and PDO on time scales of 35 years arise. With these physically explainable results, we have confidence that DPCCA is an useful method in addressing complex systems.

Complex systems, such as in climatology, ecology, economy or finance, usually contain a large number of interactions^1^,^2^,^3^,^4^. By analyzing the cross-correlations between signals observed from the complexity, one aim is to better diagnose and understand the whole system. Duo to its simplicity, traditional cross-correlation analysis (TCA) has become the most widely used method in statistics. Especially in statistical climatology, TCA is used in various fields, including dynamical diagnosing and climate forecasting^5^,^6^,^7^. However, due to the effects of many nonlinear processes and external forcings, it should be noted that signals obtained in nature are usually characterized by multi-scale structures and non-stationarities^8^,^9^. Therefore, traditional cross-correlation analysis is not always appropriate and can provide erroneous results.

For example, suppose we are interested to diagnose the relations between the Summer (June, July and August) Rainfall over the middle-lower reaches of the Yangtze River (SRYR) and the previous winter-time (December, January and February) Nino3 Sea Surface Temperature Anomaly (Nino3-SSTA). Both records range from 1951 to 2012. SRYR is calculated according to the precipitation station data provided by the Chinese National Climate Center (http://ncc.cma.gov.cn), see Figure 1, while Nino3-SSTA is downloaded from the National Oceanic & Atmospheric Administration (NOAA, http://www.esrl.noaa.gov/psd/data/climateindices/). It is a well known fact that SRYR are teleconnected with the previous winter-time East Pacific SSTA^10^,^11^, thus significant correlations are expected. However, by simply applying TCA to the two records, the calculated correlation coefficient is only 0:19, which is not statistically significant according to the student's t-test. To explain this low correlation coefficient, one reason could be that the connection between SRYR and Nino3-SSTA is nonstationary over time. Thus, it may be an unreasonable choice to analysis their relation over the whole length (1951–2012) since the climate regime is considered changed around the end of 1970s^12^. However, it may be also a “*time scale*” problem. Since Nino3-SSTA is an El Ni*ñ*o indicator with a typical period of 2 ~ 7 years, its connections with SRYR may be only significant on these time scale. On other time scales, significant correlations may disappear, which further result in a low cross-correlation coefficient calculated over the whole length. Thus, the correlations between two sub-systems can be different on different time scales.

In traditional statistics, one can apply filter methods (including low-pass filter, high-pass filter, and band-pass filter) to discuss correlations of two considered time series on different time scales^13^,^14^,^15^. However, the low (high) pass frequency, or the band-width are usually chosen subjectively, which make these simple filter methods not appropriate in performing cross-correlation research over different time scales. Another method, cross-spectral analysis (CSA)^16^,^17^, may also be useful in discussing connections of two time series on different time scales, but it requires the analyzed data to be stationary with no external trends, which of course are rare in nature. Recently, a new method based on detrended covariance, detrended cross-correlation analysis (DCCA), has been proposed and widely used^18^. DCCA is a modification of the standard covariance analysis, but can be used in the research of non-stationary time series^19^. DCCA is also a generalization of detrended fluctuation analysis (DFA)^20^,^21^, but can be used to investigate the power-law cross-correlations between *two* simultaneously recorded time series. By further calculating the DCCA cross-correlation coefficient *ρ_DCCA_* according to the procedure proposed by^22^,
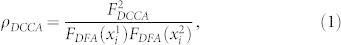
where *F_DCCA_* is the fluctuation function obtained from DCCA^18^, *F_DFA_* is the fluctuation function obtained from DFA^20^, and 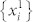, 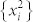 are the two considered time series, one can quantify the level of cross-correlations on different time scales. Therefore, during the past few years, signals from various fields such as economics^23^, seismic studies^24^, traffic flows^25^, as well as geophysical systems^26^, have been analyzed by using DCCA and its multifractal version MFDCCA^27^. In this study, we will mainly focus on the DCCA cross-correlation coefficient *ρ_DCCA_* derived from DCCA.

In Figure 2 (top panel), we analyze the relations between SRYR and lead Nino3-SSTA by calculating DCCA cross-correlation coefficient *ρ_DCCA_*. Apparently, SRYR is correlated with Nino3-SSTA on time scale of 5 ~ 7 years with cross-correlation coefficient larger than 0.3. While on other time scales, the cross-correlations drop to a very low level (around 0.1). This result is in line with our discussion above, and indicates that studying correlations on different time scales is very important for better understanding the whole complex system.

However, it should be further noted, that signals observed from a complex system are normally linked via interwoven heterogeneous ties. Quantifing cross-correlations between only *two* signals may be not sufficient and can provide erroneous results. Especially in the case, when the two signals are both correlated with other signals simultaneously. Such as the relations among SRYR, Nino3-SSTA, and the Pacific Decadal Oscillation (PDO). PDO is a pattern of warm or cold anomalous surface waters in north Pacific (north of 20°N), with inter-decadal time scale of about 30 years^28^. It is a well known fact that both the winter-time Nino3-SSTA and the winter-time PDO index can be considered as important precursor factors of the following summer rainfall over Yangtze River^29^,^30^. However, since PDO and El Ni*ñ*o are also coupled with each other^31^,^32^, simple analysis based on either PDO index or Nino3-SSTA may provide biased information. As shown in Figure 2 (bottom panel), we calculated DCCA cross-correlation coefficient *ρ_DCCA_* to study the relations between SRYR and PDO index. The PDO index is downloaded from the National Oceanic & Atmospheric Administration (NOAA, http://www.esrl.noaa.gov/psd/data/climateindices/), with only winter-time data selected. It is obvious that the results from PDO and that from Nino3-SSTA have similar pattern, especially on small time scale of 4 ~ 8 years (El Ni*ñ*o typical scale) and large time scale of 30 ~ 45 years (PDO typical scale), which indicates strong coupling between PDO and Nino3-SSTA. When making diagnostic analysis or prediction, one usually prefers to take as many related factors as possible into account to improve the accuracy. Here we argue that, first we need to state to what extent and on which time scales each related factor is independently affecting the system of interest, then further consider how these factors are connected to each other. One way to address this is by applying detrended partial-cross-correlation analysis (DPCCA). DPCCA is based on DCCA, thus can provide information on different time scales. Compared to DCCA cross-correlation coefficient *ρ_DCCA_*, *ρ_DPCCA_* calculated from DPCCA is further upgraded by combining partial-correlation technique, therefore it is expected to be useful in quantifing correlations of multi-signals (not only two signals) in a complex system.

In this report, we will first illustrate the advantages of DPCCA by conducting two numerical tests. Test I shows the advantages of DPCCA in handling non-stationary signals, while Test II illustrate the advantage of DPCCA in revealing “intrinsic” relations between two time series of interest, with potential influences of other unconsidered signals removed. Furthermore, the utility of DPCCA is confirmed by revisiting the climatic example mentioned above. Results and discussions are shown in the next sections. In the last part of this report, we will show explicitly how the DPCCA is designed.

## Results

### Advantages of DPCCA

Since DPCCA is based on the DCCA method but improved by combining the partial cross-correlation analysis (PCCA), it is expected to have the advantages of both methods. Therefore, we will perform two tests to verify the utility of DPCCA as indicated below.

Test I: According to^18^, DCCA is designed to investigate cross-correlations between two time series with nonstationarity. When nonstationarity such as local trends or periodic background exist, without detrending, there will be crossovers in the fluctuation function *F_DCCA_* as a function of time scale^33^,^34^, and the DCCA cross-correlation coefficient *ρ_DCCA_* calculated from Eq.(1) will be spuriously high^35^. Fortunately, by choosing an appropriate detrending order, DCCA is able to remove the effects of nonstationarity, and further provide us reliable information on the cross-correlation^36^. Similarly, DPCCA should also have this advantage. Suppose we have three independent and identically distributed (i.i.d) Gaussian variables: 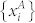, 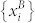 and 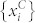 (with length of 10,000). They are not related to each other. However, if we generate another two time series: 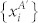 and 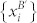, which are combinations of time series 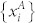 with 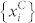 (as 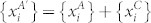), and 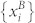 with 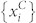 (as 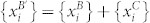), respectively, the two new generated time series will be correlated, and are both related to 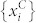. By applying the partial cross-correlation analysis to the three time series 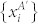, 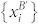 and 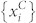, one can remove the influence of 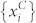 on 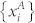 and 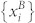, and further discover the “intrinsic” correlations between 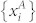 and 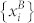 (which should be zero). However, if the three variables are all nonstationary with nonlinear trends, traditional PCCA may fail in detecting the “intrinsic” correlation information. As shown in Figure 3a, 3b, 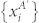 and 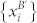 are shown with quadratic trends. The cross-correlation coefficient between 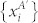 and 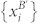 is 0.71 (Figure 3c, the blue line). After removing the influence of 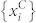, the PCCA coefficient between the two variables drops to 0.59 (the red line), which is still significantly higher than zero. If the influence of quadratic trends is removed by DCCA, the coefficient *ρ_DCCA_* further decreases to 0.51 (the yellow line), but still not the expected result. In this case, however, if we apply DPCCA, “intrinsic” relation between 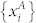 and 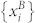 is finally obtained (the black curve in Figure 3c). In fact, not only for the case when quadratic trends exist, for cases with cubic trends, or even sinusoidal trends, DPCCA still shows reliable and accurate results, as shown in Figure 3d. We show the cases with “No Trend”, “Linear Trend”, “Quadratic Trend”, “Cubic Trend”, as well as “Sinusoidal Trend”. By applying DPCCA with appropriate detrend order (see the discussions in^36^, and also in the “Methods” section. One can remove the non-stationary effects by substracting local trends with appropriate polynomial order. Normally, DPCCAn means the polynomial order of n), expected results still arise (Black line). While other methods failed, such as PCCA (the red line) and DCCA cross-correlation coefficient *ρ_DCCA_* (the yellow line). Therefore, from this test we confirm that DPCCA has the advantages of DCCA.

Test II: Another advantage of DPCCA should originated from the partial cross-correlation analysis. Compared with the DCCA cross-correlation coefficient *ρ_DCCA_*, DPCCA can be used to investigate the correlations of multi-signals in a complex system, and find the “intrinsic” relations between two considered signals. Suppose we have two independent and identically distributed (i.i.d) Gaussian variables: 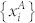 and 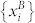 (with length of 10,000). By adding sinusoidal signals 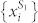 and 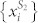 (as shown in Figure 4a–b), two new time series 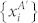 and 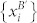 can be generated, as 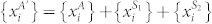, and 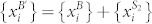. The two sinusoidal signals have different frequencies, as shown in Figure 4, 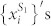 cycle is 1000 (days), while 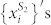 cycle is 100 (days). Therefore, in the newly generated time series, 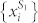 can be considered as a background field, and the 100 (days) periodic signal (corresponding to 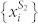) can be detected by DCCA cross-correlation coefficient *ρ_DCCA_*, as shown in Figure 4e (the red line). However, if another sinusoidal signal 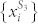 (as shown in Figure 4c, which has the same frequency with 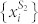, but with different phases), is also added to 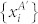, by simply calculating DCCA cross-correlation coefficient *ρ_DCCA_*, one may underestimate the 100 (days) periodic signal due to the “offset” effect between 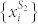 and 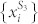 (Figure 4d and Figure 4e, the open circles). Therefore, in this case, *ρ_DPCCA_* should be a more appropriate choice for our analysis and diagnose. See Figure 4f, by applying DPCCA to the three time series 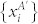, 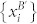, and 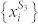, we can remove the influence of 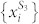 on 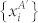 successfully, and reveal the 100 (days) periodic signal accurately. Therefore, from this test we confirm that DPCCA inherits the advantages of the partial-correlation technique.

### Application of DPCCA to natural complex system

Considering natural signals are normally recorded from complex systems, they are usually characterized by non-stationary, and are always correlated with other multi-signals. Therefore, the DPCCA method proposed in this report could be widely used in various fields. In the following, we will further illustrate the utility of DPCCA by revisiting the climatic example we have mentioned in the introduction.

**Figure 2:**
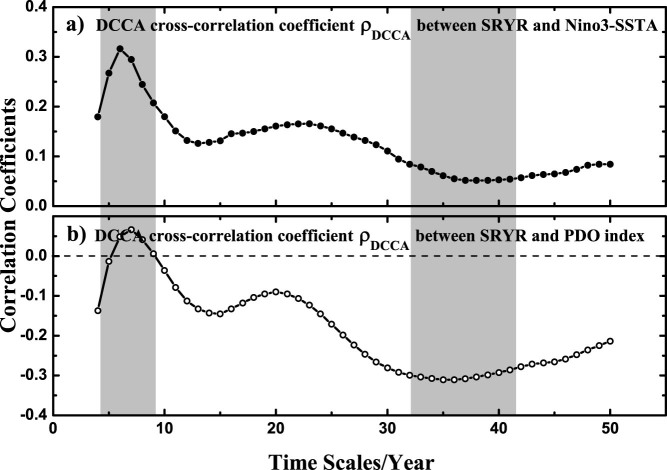
DCCA cross-correlation coefficient *ρ_DCCA_* between SRYR and Nino3-SSTA (a), as well as SRYR and PDO index (b). The grey region covers the time scale of 5 ~ 8 years (El Ni*ñ*o typical scale) and the time scale of 33 ~ 42 years (PDO typical scale). The two curves have similar pattern.

**Figure 3:**
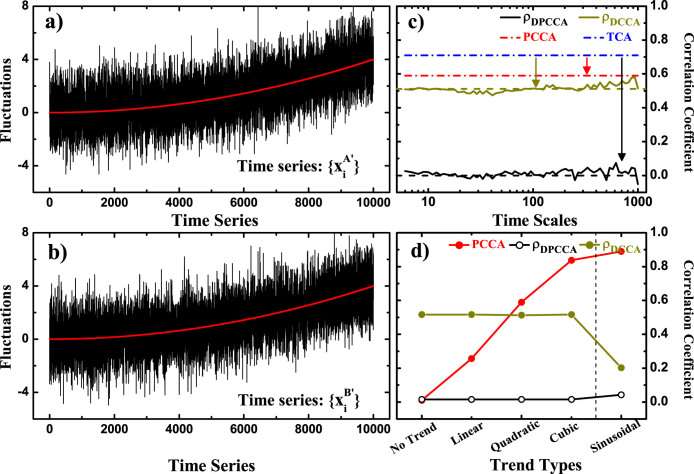
Time series and related results in Test I. (a, b) show the two time series 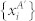 and 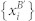 in test I, but with quadratic trends. The results of traditional cross-correlation analysis (TCA, blue), partial cross-correlation analysis (PCCA, red), detrended cross-correlation analysis (DCCA cross-correlation coefficient *ρ_DCCA_*, yellow), as well as the detrended partial-cross-correlation analysis (DPCCA, black) are shown in (c). In (d), more cases with higher-order trends are shown. For each “Trend Type”, the detrend order of DPCCA (DCCA) are different, as DPCCA1 (DCCA1), DPCCA2 (DCCA2), DPCCA3 (DCCA3), DPCCA4 (DCCA4), and DPCCA16 (DCCA16), respectively. The coefficient of each “Trend Type” is actually the mean correlation coefficient averaged over all the time scales in (c).

We study how the winter-time Pacific Decadal Oscillation (PDO) and winter-time Nino3 Sea Surface Temperature Anomaly (Nino3-SSTA) affect the Summer Rainfall over the middle-lower reaches of the Yangtze River (SRYR) over the past 60 years. It has been well recognized that the summer rainfall over China is influenced by two main modes of Pacific SST variation: PDO and El Ni*ñ*o (with Nino3-SSTA as an indicator^37^). Their winter signals are both considered as important precursor factors of the summer rainfall over China^29^,^30^. However, since PDO and El Ni*ñ*o are also coupled with each other^31^,^32^ (Figure 2), simple predictions based on either PDO or Nino3-SSTA are not entirely reliable. Therefore, we need to reveal the “intrinsic” relations between SRYR and PDO, as well as the “intrinsic” relations between SRYR and Nino3-SSTA. Figure 5 shows the results, where significant differences between the output of DPCCA and DCCA are presented. For the relations between SRYR and Nino3-SSTA, after removing the influence of PDO, much higher (positive) cross-correlation coefficients *ρ_DPCCA_* over all the time scales are found. Especially on time scales of 5 ~ 8 years (the gray area), more significant cross-correlations between SRYR and Nino3-SSTA are found (exceeding the 95% confidence level), which corresponds to the typical period of El Ni*ñ*o. As for the relations between SRYR and PDO, after removing the influence of Nino3-SSTA, much lower (negative) cross-correlation coefficients *ρ_DPCCA_* over all time scales are obtained. If we calculate DCCA cross-correlation coefficient *ρ_DCCA_* only, positive correlations between SRYR and PDO on time scales of 6 ~ 8 years are found, however not significant. After removing the effect of Nino3-SSTA, the positive correlations disappear. Interestingly, on time scales of about 35 years (the grey area), significant (negative) correlations between SRYR and PDO arise (exceeding the 95% confidence level), which corresponds to the typical period of PDO. However, masked by the El Ni*ñ*o, this signal cannot be revealed from *ρ_DCCA_*. From these results it becomes obvious that El Ni*ñ*o has important impacts on SRYR during its typical period (5 ~ 8 years), while at the multidecadal scale, the SRYR may be modulated by the PDO. This finding is in line with previous studies. In fact, it has been well accepted that during the period of El Ni*ñ*o, a persistent anomalous anticyclone over the Western North Pacific (WNP) can bring a large amount of water vapor to East Asia, which leads to an increase of precipitation over the Yangtze River^38^,^39^,^40^,^41^. However, modulated by the locations and strengths of WNP monsoon trough and the WNP subtropical high (WNPSH)^41^,^42^, which maybe related to the variations of PDO, the effects of El Ni*ñ*o on East Asia can also vary on multidecadal scale. Such as the time before the late 1970s, positive (negative) winter-time Nino3-SSTA usually corresponds to less (more) rainfall over the Yangtze River. Due to a westward expansion of the WNPSH after late 1970s, summer precipitation increased over the Yangtze River^43^,^44^. Therefore, for better understanding the Summer Rainfall over the middle-lower reaches of the Yangtze River, different mechanisms on different time scales should be considered carefully. From this example, cross-correlation coefficient *ρ_DPCCA_* obtained from DPCCA shows better performance than *ρ_DCCA_* from DCCA.

## Discussion

**Figure 4:**
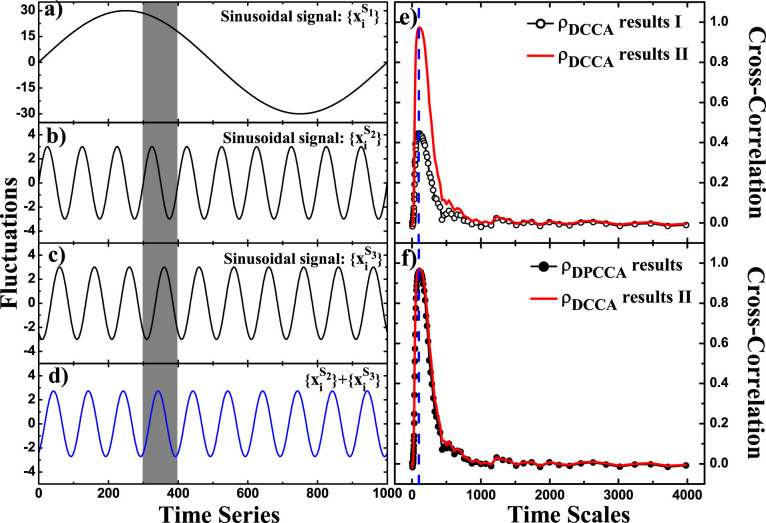
Time series and related results in Test II. (a–c) show fractions of the three sinusoidal signals in test II: 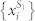, 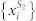, and 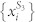. 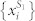 acts as a background field, with low-varying frequency and larger amplitude. 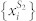, and 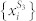 have different phases (see the gray part), and their combination is shown in (d). The red curve in (e) is the DCCA cross-correlation coefficient *ρ_DCCA_* between 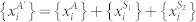 and 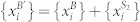 (denoted as *ρ_DCCA_* results II), and the blue dashed line shows the time scale of 100 (days). If the signal 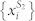 in 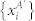 is offset by 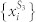, *ρ_DCCA_* fails in providing accurate results, as shown in (e), the black open-circle curve (denoted as *ρ_DCCA_* results I). But DPCCA succeeds, as shown in (f), the black solid-circle curve.

In this report, we proposed a new method, Detrended Partial-Cross-Correlation Analysis (DPCCA), which can be used to diagnose “intrinsic” relations of two nonstationary signals (with influences of other signals removed) on different time scales. This method is based on the Detrended Cross-Correlation Analysis (DCCA), but improved by including the Partial-Cross-Correlation Analysis (PCCA), which therefore has the advantages of both DCCA and PCCA. To illustrate the advantages, we made two simple tests in our study. Test I proved DPCCA indeed can provide robust results even when nonlinear trends are mixed in the data we are analyzing, and further show relations between the two considered data on different time scales. While Test II illustrated the ability of DPCCA in investigating correlations when multi-signals are linked via interwoven ties, as shown in Figure 4. In general, DPCCA has better performance in dealing with correlations in complex system. However, when applying it, there are two points that need to be considered.

**Figure 5:**
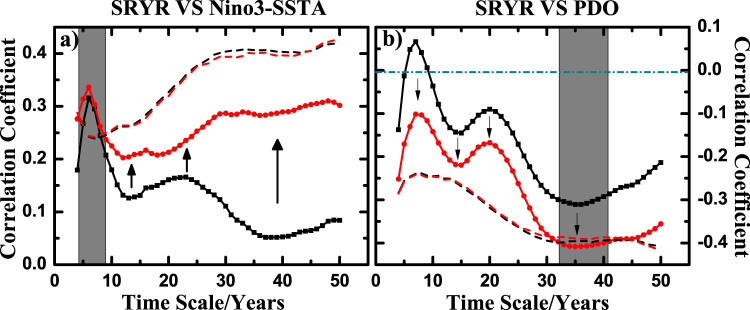
Application of DPCCA to the climatic example. DCCA (black) and DPCCA (red) outputs between (a) SRYR and Nino3-SSTA, (b) SRYR and PDO. The black dashed line represents the 95% significance criterions of *ρ_DCCA_* on different time scales, while the red dashed line represents the criterions of *ρ_DPCCA_*. They are both obtained from Monte-Carlo Simulations according to^45^. We shuffled the considered time series and repeated the DCCA/DPCCA calculations for 10,000 times. The top 2.5% largest values (on different time scales) give the dashed lines in (a), while the top 2.5% smallest values give the dashed lines in (b). The grey area covers the typical time scales where (a) the Nino3-SSTA has significant positive correlation with SRYR on time scales of 5 ~ 8 years, and (b) the PDO has significant negative correlation with SRYR on time scales of about 35 years.

 i) Significance testing. With DPCCA, one can obtain cross-correlations on different time scales. However, to determine whether the calculated correlations are statistically significant, one can not simply apply the student's t-test due to the changing degree of freedom. Normally, Monte-Carlo tests have to be applied to decide whether the obtained cross-correlations are significant on a given time scale^45^ (Figure 5, the blue line).

 ii) Background assumptions. When applying DPCCA, one has to pay attention to the background assumptions of partial cross-correlation analysis. That is, the considered multi-signals should have linear relationships with each other. This is the main deficiency of PCCA. However, by using DCCA, we believe this deficiency can be reduced to some extent, since only the relationships on different time scales are discussed, but not on the whole length. Nevertheless, we would like to stress that more advanced analytical methods are needed, especially the methods based on nonlinear frameworks.

We applied DPCCA to a climatic example, that deals with the winter-time Pacific Decadal Oscillation (PDO) and the winter-time Nino3 Sea Surface Temperature Anomaly (Nino3-SSTA) affecting the Summer Rainfall around Yangtze River (SRYR) over past decades. Since PDO has longer variation period (≈30 years), it can be considered as a variable background. With PDO controlled, the relations between Nino3-SSTA and SRYR seems to be more apparent on the time scales of 5 ~ 8 years. Similarly, with Nino3-SSTA controlled, significant relations between PDO and SRYR emerges on time scales of about 35 years. Although, due to possible influences of nonlinear effects, our results may still be problematic, considering traditional Cross-Correlation Analysis is still the main analytical method in various fields, our study still improves our ability in analyzing cross-correlations among multi-variables on different time scales. From the two numerical tests and the climatic example, we can summarize the advantages of DPCCA. i) DPCCA can be used to reveal the “intrinsic” relations between two considered variables, by removing the possible influences of other unconsidered signals, ii) DPCCA is appropriate in the research of non-stationary variables, and iii) DPCCA can show the correlation levels on different time scales. Based on these advantages, we are convinced that this method will have extensive application prospects.

## Methods

In this section, we will show the details on how the method, DPCCA, is designed.

Suppose we have *m* time series 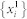, 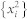, 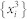, 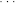, 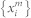, where 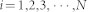. Each time series can be considered as a random walk, and we can define the so called profile as:
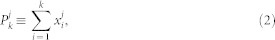
where 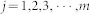, 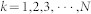. Similar to the procedures in DCCA, one first divide the entire profile into *N* − *s* overlapping boxes. Each box *i* contains *s* + 1 values, starts at *i* and ends at *i* + *s*. In each box *i*, we can determine the “local trend” 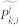 (*i* ≤ *k* ≤ *i* + *s*) by using a polynomial fit, and further define the “detrended walk” as the difference between the original profile and the local trend, as:
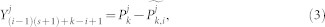
In this way, we can get one detrended residual series 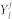, 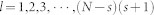, for each time series 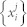. By calculating the covariance between any two residuals,
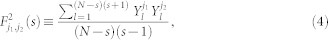
where 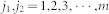, we can obtain a covariance matrix,
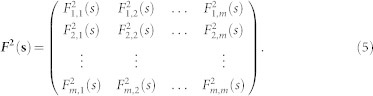
Obviously, according to^22^, the cross-correlation levels between any two time series, 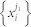 and 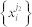, can be estimated as,
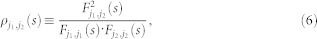
and a coefficients matrix can further be obtained as,
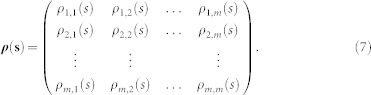
Where 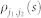 ranges from −1 to +1, and represents the level of cross-correlation on time scales of *s*. However, it should be noted that it only shows the relations between time series 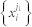 and 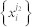. This may provide spurious correlation information if the two time series are both correlated with other signals. Therefore, to exclude the possible influence of other time series, we need to combine the partial-correlation technique with the calculations above.

To apply the partial-correlation technique, we first need to calculate the inverse matrix of ***ρ***(***s***),
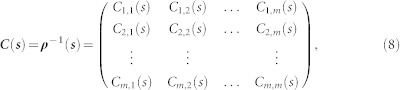
for any two time series 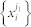 and 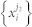, the partial-cross-correlation level can thus be determined as,
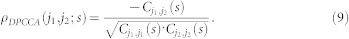
where the coefficients *ρ_DPCCA_*(*j*_1_, *j*_2_; *s*) can be used to characterize the “intrinsic” relations between the two time series on time scales of *s*. It is worth to note that we use the word “intrinsic” here, is to indicate a condition when the influences of other time series have been removed, or assume a situation that other time series remain unchanged. By changing *s*, similar to the DCCA cross-correlation coefficient *ρ_DCCA_*, we can further estimate the partial cross-correlation levels on different time scales.

## Author Contributions

N.Y. designed the study, N.Y. and Z.F. performed the study. N.Y., H.Z., L.P., J.L. and E.X. wrote the main manuscript. All the authors reviewed the manuscript.

## Figures and Tables

**Figure 1:**
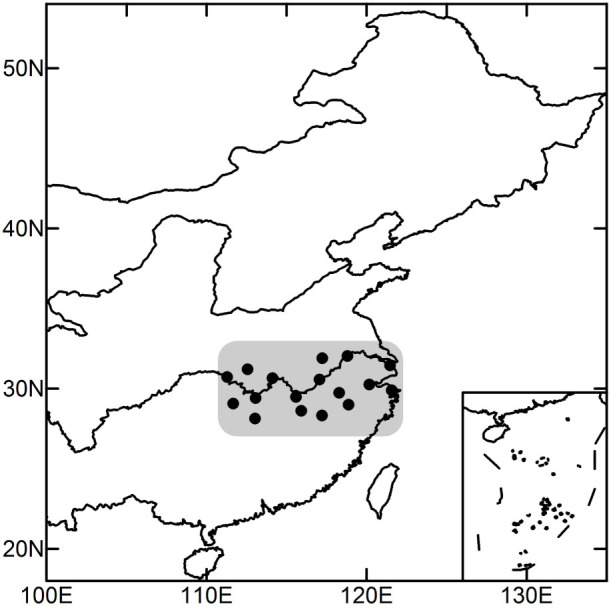
Geographic locations of the stations around the Yangtze River. 17 stations over the middle-lower reaches of the Yangtze River are chosen for the calculation (average over the 17 stations) of SRYR. Their locations are shown as the solid circles. We generate the figure by using Surfer 8.0 (Golden Software, http://www.goldensoftware.com/products/surfer).
